# Current status of surgery for clinical stage IA lung cancer in Japan: analysis of the national clinical database

**DOI:** 10.1007/s00595-020-02063-x

**Published:** 2020-07-05

**Authors:** Norihiko Ikeda, Shunsuke Endo, Eriko Fukuchi, Jun Nakajima, Kohei Yokoi, Masayuki Chida, Hiroshi Date, Akinori Iwasaki, Hiroyasu Yokomise, Masami Sato, Meinoshin Okumura, Hiroyuki Yamamoto, Hiroaki Miyata, Takashi Kondo

**Affiliations:** 1 Department of Surgery, Tokyo Medical University, 6-7-1 Nishishinjuku, Shinjuku-ku, Tokyo, 160-0023 Japan; 2 Department of Thoracic Surgery, Jichi Medical University, 3311-1 Yakushiji, Shimotsuke-shi, Tochigi 329-0498 Japan; 3 Department of Healthcare Quality Assessment, The University of Tokyo, 7-3-1 Hongo, Bunkyo-ku, Tokyo, 113-8655 Japan; 4 Department of Thoracic Surgery, Graduate School of Medicine, The University of Tokyo, 7-3-1 Hongo, Bunkyo-ku, Tokyo, 113-8655 Japan; 5 Department of Thoracic Surgery, Nagoya University Graduate School of Medicine, 65 Tsurumai-cho, Showa-ku, Nagoya, Aichi 466-8550 Japan; 6 Department of General Thoracic Surgery, Dokkyo Medical University, 880 Kitakobayashi, Mibu-machi, Shimotsuga-gun, Tochigi 321-0293 Japan; 7 Department of Thoracic Surgery, Kyoto University Graduate School of Medicine, 54 Shogoin-Kawara-cho, Sakyo-ku, Kyoto City, Kyoto 606-8507 Japan; 8 Department of General Thoracic, Breast, and Pediatric Surgery, Faculty of Medicine, Fukuoka University, 7-45-1 Nanakuma, Jonan-ku, Fukuoka City, Fukuoka 814-0180 Japan; 9 Department of General Thoracic Surgery, Breast and Endocrinological Surgery, Faculty of Medicine, Kagawa University, 1750-1 Ikenobe, Miki-cho, Kita-gun, Kagawa 761-0793 Japan; 10 Department of General Thoracic Surgery, Graduate School of Medical and Dental Sciences, Kagoshima University, 8-35-1 Sakuragaoka, Kagoshima City, Kagoshima 890-8544 Japan; 11 Department of General Thoracic Surgery, Graduate School of Medicine, Osaka University, 2-15 Yamadaoka, Suita, Osaka 565-0871 Japan; 12 Department of Thoracic Surgery, Tohoku Medical and Pharmaceutical University, 1-12-1 Fukumuro, Miyagino-ku, Sendai, Miyagi 983-8512 Japan

**Keywords:** Lung cancer, Surgery, National database, VATS

## Abstract

**Purpose:**

As the number of cases of early lung cancer in Japan grows, an analysis of the present status of surgical treatments for clinical stage IA lung cancer using a nationwide database with web-based data entry is warranted.

**Methods:**

The operative and perioperative data from 47,921 patients who underwent surgery for clinical stage IA lung cancer in 2014 and 2015 were obtained from the National Clinical Database (NCD) of Japan. Clinicopathological characteristics, surgical procedure, mortality, and morbidity were analyzed, and thoracotomy and video-assisted thoracic surgery (VATS) were compared.

**Results:**

The patients comprised 27,208 men (56.8%) and 20,713 women (43.2%); mean age, 69.3 years. Lobectomy was performed in 64.8%, segmentectomy in 15.2%, and wedge resection in 19.8%. The surgical procedures were thoracotomy in 12,194 patients (25.4%) and a minimally invasive approach (MIA) in 35,727 patients (74.6%). MIA was divided into VATS + mini-thoracotomy (*n* = 13,422, 28.0%) and complete VATS (*n* = 22,305, 46.5%). The overall postoperative mortality rate was 0.4%, being significantly lower in the MIA group than in the thoracotomy group (0.3% vs 0.8%, *P* < 0.001).

**Conclusions:**

Our analysis of data from the NCD indicates that MIA has become the new standard treatment for clinical stage IA lung cancer.

**Electronic supplementary material:**

The online version of this article (10.1007/s00595-020-02063-x) contains supplementary material, which is available to authorized users.

## Introduction

Lung cancer is the leading cause of cancer-related deaths across the world. The 5-year survival rate of stage IA lung cancer patients is 86.8% [1]; therefore, curative treatment in the early stage is indicative of a favorable prognosis. Video-assisted thoracic surgery (VATS) is used widely for the treatment of stage IA lung cancer. In Japan, 28,568 lung cancer operations were performed using VATS in 2016, accounting for 67.8% of the total operations (*n* = 42,107) [2]. Thus, an assessment of the surgical treatments for stage I lung cancer will be very useful for clarifying the present status of chest surgery and VATS.

The National Clinical Database (NCD) of Japan is a nationwide web-based surgical patient registration system that enables the collection of data on surgical procedures and perioperative factors as part of the surgical specialization by the Japanese Surgical Board Certification System [3, 4]. In 2014, the data on 1.6 million surgical procedures from more than 4000 hospitals were collected [3]. A committee authorized by the NCD evaluates and compares the reliability of samples and the web-based data from the NCD. The results indicate a high percentage of data correctness (> 94%) even in the initial year of data collection [5]. To ensure the accuracy and traceability of data, the NCD continuously tracks authorized persons responsible for data entry through the web-based data management system in combination with random site visits.

The NCD for chest surgery requires the input of detailed data on patient characteristics, operative information, and postoperative events (Supplemental Table 1) [5]. The NCD was used to analyze cases of stage IA lung cancer (UICC-TNM version 7) to obtain a representative picture of the surgical treatments for early stage lung cancer in Japan, as well as to compare thoracotomy and minimally invasive approach (MIA) surgery with extensive use of VATS.

## Methods

The study population was derived from the NCD data on patients who underwent surgery for primary lung cancer between January 1, 2014 and December 31, 2015, at 887 surgical units. Records with incomplete data or unspecified patient status within 30 days after surgery were excluded. A total of 78,594 patients who underwent lung cancer resection, with complete data, were registered. Patients with clinical stage IB-IV (*N* = 30,590) and those who underwent robotic surgery (*N* = 83) were excluded. Finally, 47,921 patients with surgically treated clinical stage IA lung cancer were selected for analysis.

The NCD registry required the selection of either thoracotomy or VATS as the surgical approach for each lung cancer operation. Herein, we reclassified the surgical approach used in registered cases into thoracotomy or MIA. MIA was divided into complete VATS and VATS with mini-thoracotomy of 8 cm or less (VATS + mini-thoracotomy) [6] according to the definition of Swanson et al. [7]. The presence or absence of rib-spreading is not yet specified in the NCD registry.

Accordingly, the comorbidities should be entered in accordance with the established criteria [5]. The surgical characteristics were analyzed in terms of procedure, type of nodal dissection, blood loss, number of staples applied, maximum wound length, conversion to thoracotomy, and number of access ports [5]. Postoperative major morbidity was defined in accordance with the Society of Thoracic Surgeons risk model [6, 8, 9].

### Statistical analysis

The Chi-square test and Fisher’s exact test were used to compare categorical data and their distributions, as appropriate. All *P* values were two-sided, and *P* < 0.05 was considered to indicate a statistically significant difference. For continuous data, Student’s *t* test was performed for the comparison of two groups, and one-way ANOVA followed by Bonferroni analysis was performed for the comparison of three groups. For non-normally distributed continuous data, we used the Wilcoxon rank-sum test and the Kruskal–Wallis test, as appropriate. Statistical analysis was performed using the SPSS statistical software package (Version 24.0; SPSS Inc., Chicago, IL) and STATA 16 software (Stata, College Station, Texas). This study was approved by the Ethics Committee of Tokyo Medical University (T2018-0018).

## Results

Table 1 summarizes the clinical characteristics of the registered patients. There were 27,208 men (56.8%) and 20,713 women (43.2%); mean age, 69.3 years. The disease was classified as clinical T1a (62%) or T1b (38%). The surgical approach was thoracotomy in 12,194 patients (25.4%) and MIA in 35,727 patients (74.6%). MIA was divided into VATS + mini-thoracotomy (*n* = 13,422, 28.0%) and complete VATS (*n* = 22,305, 46.5%).

**Table 1:** Clinical characteristics of the patients

	Total	Thoracotomy	MIA	*P *value (Thoracotomy vs MIA)	MIA	
					VATS + mini-thoracotomy	Complete VATS
No. of patients	47,921	12,194 (25.4%)	35,727 (74.6%)		13,422 (28.0%)	22,305 (46.5%)
Age (years) Mean ± SD	69.3 ± 9.3	69.6 ± 8.8	69.2 ± 9.5	< 0.001	69.4 ± 9.5	69.0 ± 9.6
Sex (Male/Female)	27,208 (56.8%)/20,713(43.2%)	7614 (62.4%)/4580 (37.6%)	19,594 (54.8%)/16,133 (45.2%)	< 0.001	7378 (55.0%)/6044 (45.0%)	12,216 (54.8%)/10,089 (45.2%)
clinical T status				< 0.001		
cT1a	29,670 (61.9%)	6847 (56.2%)	22,823 (63.9%)		8442 (62.9%)	14,381 (64.5%)
cT1b	18,250 (38.1%)	5347 (43.8%)	12,903 (36.1%)		4980 (37.1%)	7923 (35.5%)
cTis	1 (0.0%)	0 (0.0%)	1 (0.0%)			
Tumor diameter (cm) Mean ± SD	1.85 ± 0.61	1.94 ± 0.60	1.82 ± 0.61	< 0.001	1.84 ± 0.60	1.81 ± 0.61
Comorbidities						
Diabetes mellitus^a^	6566 (13.7%)	1707 (14.0%)	4859 (13.6%)	0.272	1782 (13.3%)	3077 (13.8%)
Ischemic heart disease^b^	2569 (5.4%)	657 (5.4%)	1912 (5.4%)	0.872	670 (5.0%)	1242 (5.6%)
Arrhythmia^c^	1719 (3.6%)	450 (3.7%)	1269 (3.6%)	0.482	426 (3.2%)	843 (3.8%)
Interstitial pneumonia^d^	1757 (3.7%)	524 (4.3%)	1233 (3.5%)	< 0.001	449 (3.3%)	784 (3.5%)
Cerebral nervous disorder^e^	2714 (5.7%)	675 (5.5%)	2039 (5.7%)	0.496	755 (5.6%)	1284 (5.8%)
Liver dysfunction^f^	221 (0.46%)	50 (0.41%)	171 (0.48%)	0.353	71 (0.53%)	100 (0.45%)

*MIA* minimally invasive approach

^a^Required treatment or supplementary explanation

^b^Required treatment or had previous treatment

^c^Required treatment

^d^Interstitial pneumonia shadow on chest computed tomography

^e^Required treatment or had previous treatment

^f^Child-Turcotte classification B or C

The tumor diameter (mean ± standard deviation) was 1.85 ± 0.61 cm overall, 1.94 ± 0.60 cm in the thoracotomy group, and 1.82 ± 0.61 cm in the MIA group (*P* < 0.001). The comorbidities included diabetes mellitus (13.7%), cerebral nervous system disorders (5.7%), ischemic heart disease (5.4%), and interstitial pneumonitis (3.7%). The rates of preoperative comorbidities, apart from interstitial pneumonitis, were not significantly different between the thoracotomy group and the MIA group.

Table 2 shows the results of pathological examination. Adenocarcinoma was the most frequent histologic type (76.3%), followed by squamous cell carcinoma (13.8%). Pathological IA cancer was observed more frequently in the MIA group (79.2%) than in the thoracotomy group (73.1%); however, pathological II and IIIA disease (up-staging) was more frequent in the thoracotomy group. Among the 393 patients with stage IV disease, 359 were diagnosed as having M1a with pleural dissemination and/or pulmonary metastasis which could not be detected preoperatively.

**Table 2:** Pathological characteristics of the patients

	Total	Thoracotomy	MIA	*P *value (Thoracotomy vs MIA)	MIA	
					VATS + mini-thoracotomy	Complete VATS
Histology				< 0.001		
Adenocarcinoma	36,570 (76.3%)	8848 (72.6%)	27,722 (77.6%)		10,370 (77.3%)	17,352 (77.8%)
Squamous cell carcinoma	6603 (13.8%)	1974 (16.2%)	4629 (13.0%)		1778 (13.2%)	2851 (12.8%)
Large cell carcinoma	743 (1.6%)	232 (1.9%)	511 (1.4%)		179 (1.3%)	332 (1.5%)
Small cell carcinoma	785 (1.6%)	244 (2.0%)	541 (1.5%)		219 (1.6%)	322 (1.4%)
Adenosquamous carcinoma	500 (1.0%)	148 (1.2%)	352 (1.0%)		134 (1.0%)	218 (1.0%)
Others	2720 (5.7%)	748 (6.1%)	1972 (5.5%)		742 (5.5%)	1230 (5.5%)
Pathological stage				< 0.001		
0	519 (1.1%)	77 (0.6%)	442 (1.2%)		164 (1.2%)	278 (1.2%)
IA	37,195 (77.6%)	8909 (73.1%)	28,286 (79.2%)		10,561 (78.7%)	17,725 (79.5%)
IB	5639 (11.8%)	1638 (13.4%)	4001 (11.2%)		1537 (11.5%)	2464 (11.0%)
IIA	1728 (3.6%)	624 (5.1%)	1104 (3.1%)		405 (3.0%)	699 (3.1%)
IIB	589 (1.2%)	206 (1.7%)	383 (1.1%)		169 (1.3%)	214 (1.0%)
IIIA	1714 (3.6%)	608 (5.0%)	1106 (3.1%)		435 (3.2%)	671 (3.0%)
IIIB	20 (0.04%)	7 (0.06%)	13 (0.04%)		5 (0.04%)	8 (0.04%)
IV	393 (0.8%)	99 (0.8%)	294 (0.8%)		116 (0.9%)	178 (0.8%)
Other	124 (0.3%)	26 (0.2%)	98 (0.3%)		30 (0.2%)	68 (0.3%)

*MIA* minimally invasive approach

Table 3 summarizes the surgical procedures. Lobectomy was performed in 64.8% of patients, segmentectomy in 15.2%, and wedge resection in 19.8%. Lobectomy was performed more frequently in the thoracotomy group (71.3%) than in the MIA group (62.6%) (*P* < 0.001). Sleeve lobectomy was performed in 25 patients and primary lesions were considered central type tumors (19 and 4 for pathological stages I and II; 12 and 6 for squamous cell carcinoma and carcinoid, respectively). Pneumonectomy was performed in 49 patients, including 10 (20.4%) with pathological stages III and IV disease and 4 (8.2%) with injury of the pulmonary artery (data not shown). The operation time (mean ± standard deviation) for all patients was 184 ± 83 min. It was significantly shorter in the MIA group (177 ± 81 min) than in the thoracotomy group (206 ± 84 min) (*P* < 0.001). The intraoperative blood loss (median) was 30 ml, being 55 ml in the thoracotomy group and 20 ml in the MIA group (*P* < 0.001). The number of staples used was 5.3 ± 2.3 overall, being 5.3 ± 2.3 in both the thoracotomy and MIA groups. MIA was converted to thoracotomy in 350 of 35,727 patients (1.0%), from VATS + mini-thoracotomy in 287 (2.1%) and from complete VATS in 63 (0.3%). The median postoperative hospital stay was 9 days in the thoracotomy group and 8 days in the MIA group (*P* < 0.001). Details about postoperative pain medication and the length of ICU stay were not registered in the NCD.

**Table 3:** Surgical characteristics

	Total	Thoracotomy	MIA	*P *value (Thora cotomy vs MIA)	MIA	
					VATS + mini thoracotomy	Complete VATS
Surgical procedure				< 0.001		
Wedge resection	9502 (19.8%)	1309 (10.7%)	8193 (23.0%)		2885 (21.5%)	5308 (23.8%)
Segmentectomy	7287 (15.2%)	2121 (17.4%)	5166 (14.5%)		2142 (16.0%)	3024 (13.6%)
Lobectomy	31,058 (64.8%)	8699 (71.3%)	22,359 (62.6%)		8389 (62.5%)	13,970 (62.6%)
Sleeve resection	25 (0.05%)	20 (0.16%)	5 (0.01%)		4 (0.03%)	1 (0.004%)
Pneumonectomy	49 (0.1%)	45 (0.37%)	4 (0.01%)		2 (0.01%)	2 (0.009%)
Postoperative hospital stay (day) median, IQR	8 (6–12)	9 (7–13)	8 (6–11)	< 0.001	9 (7–12)	8 (6–11)
Ope time (min) mean ± SD	184 ± 83	206 ± 84	177 ± 81	< 0.001	174 ± 77	179 ± 83
Blood loss (ml) median, IQR	30 (10–99)	55 (20–143)	20 (10–75)	< 0.001	27 (10–75)	20 (10–75)
No. of staples mean ± SD	5.3 ± 2.3	5.3 ± 2.3	5.3 ± 2.3	0.316	5.2 ± 2.2	5.4 ± 2.3
Maximum length of wound (cm) mean ± SD	NA	12.8 ± 4.3	4.6 ± 2.0	< 0.001	6.2 ± 1.8	3.5 ± 1.2
Conversion to thoracotomy	NA	NA	350 (1.0%)		287 (2.1%)	63 (0.3%)
No. of ports	NA	NA	3.0 ± 0.84		2.4 ± 0.79	3.3 ± 0.70

*MIA *minimally invasive approach, *IQR *interquartile range, *Ope time *operation time

* vs **: *P* < 0.001

Table 4 lists the intraoperative injuries and postoperative complications. Pulmonary artery injury occurred in 409 patients (0.85%): in 1.9% of those undergoing thoracotomy and 0.48% of those undergoing MIA (*P* < 0.001). The most frequent postoperative complications were prolonged air leakage (3.6%), pneumonia (1.4%), and arrhythmia (1.3%). Postoperative acute exacerbation of interstitial pneumonia occurred in 180 patients (0.38%), after thoracotomy in 0.57%, and after MIA in 0.31%. Major morbidity was observed in 1978 patients (4.1%): 741 (6.1%) from the thoracotomy group and 1237 (3.5%) from the MIA group (*P* < 0.001). Operative mortality was 0.4%, being significantly higher in the thoracotomy group (0.8%) than in the MIA group (0.3%) (*P* < 0.001).

**Table 4:** Major complications and mortality

	Total	Thoracotomy	MIA	*p* value (Thoracotomy vs MIA)	MIA	
					VATS + mini-thoracotomy	Complete VATS
Intraoperative injury						
Aorta	6 (0.01%)	4 (0.03%)	2 (0.006%)	0.040	0 (0%)	2 (0.009%)
Pulmonary artery	409 (0.85%)	236 (1.9%)	173 (0.48%)	< 0.001	74 (0.55%)	99 (0.44%)
Pulmonary vein	75 (0.16%)	41 (0.34%)	34 (0.10%)	< 0.001	12 (0.09%)	22 (0.10%)
Superior vena cava	10 (0.02%)	5 (0.04%)	5 (0.01%)	0.136	0 (0%)	5 (0.02%)
Postoperative complications						
Respiratory failure^a^	154 (0.3%)	72 (0.6%)	82 (0.2%)	< 0.001	41 (0.31%)	41 (0.18%)
Pneumonia	671 (1.4%)	292 (2.4%)	379 (1.1%)	< 0.001	124 (0.92%)	273 (1.2%)
Acute exacerbation of interstitial pneumonia	180 (0.38%)	70 (0.57%)	110 (0.31%)	< 0.001	47 (0.35%)	63 (0.28%)
Prolonged air leakage^b^	1720 (3.6%)	523 (4.3%)	1197 (3.4%)	< 0.001	422 (3.1%)	775 (3.5%)
Pyothorax	258 (0.5%)	92 (0.8%)	166 (0.5%)	< 0.001	71 (0.52%)	95 (0.43%)
Broncho pleural fistula	74 (0.15%)	26 (0.2%)	48 (0.1%)	0.058	12 (0.09%)	36 (0.2%)
Pulmonary embolus	30 (0.06%)	12 (0.1%)	18 (0.05%)	0.090	3 (0.02%)	15 (0.07%)
Cardiac failure^c^	63 (0.13%)	31 (0.25%)	32 (0.09%)	< 0.001	18 (0.13%)	14 (0.06%)
Arrhythmia^d^	619 (1.3%)	222 (1.8%)	397 (1.1%)	< 0.001	124 (0.9%)	273 (1.2%)
Myocardial infarction	17 (0.04%)	8 (0.07%)	9 (0.03%)	0.051	3 (0.02%)	6 (0.03%)
Cerebral infarction, hemorrhage	148 (0.31%)	48 (0.39%)	100 (0.28%)	0.056	38 (0.28%)	62 (0.28%)
Chylothorax	268 (0.56%)	69 (0.57%)	199 (0.56%)	0.893	71 (0.51%)	128 (0.57%)
Re-operation^e^	73 (0.15%)	23 (0.19%)	50 (0.14%)	0.228	25 (0.19%)	25 (0.11%)
Mortality						
Major morbidity	1978 (4.1%)	741 (6.1%)	1237 (3.5%)	< 0.001	459 (3.4%)	778 (3.5%)
Operative death (in hospital or within 30 days postop)	206 (0.4%)	94 (0.8%)	112 (0.3%)	< 0.001	49 (0.37%)	63 (0.28%)
Operative death or major morbidity	2048 (4.3%)	773 (6.3%)	1275 (3.6%)	< 0.001	479 (3.6%)	796 (3.6%)

MIA: Minimally invasive approach

^a^Required ventilation support > 48 h

^b^Air leakage > 6 days or required treatment

^c^Required treatment

^d^Required treatment (Detailed definitions are found in Ref. 5)

^e^Redo surgery within 24 h after the initial surgery

Table 5 analyzes the patients who underwent lobectomy. Of the total 31,058 patients who underwent lobectomy, 8699 (28.0%) underwent thoracotomy, 8389 (27.0%) underwent VATS + mini-thoracotomy, and 13,970 (45.0%) underwent complete VATS. The mean operation time for thoracotomy (214 ± 82 min) was significantly longer than that for VATS + mini-thoracotomy (195 ± 71 min) and that for complete VATS (208 ± 70 min) (both *P* < 0.001). The rate of conversion to thoracotomy was significantly lower in complete VATS (0.31%) than in VATS + mini-thoracotomy (2.3%) (*P* < 0.001). The number of staples used during the operation was 5.6 ± 2.0 in VATS + mini-thoracotomy and 5.9 ± 2.2 in complete VATS, the latter number being significantly greater than that in thoracotomy (5.5 ± 2.2, *P* < 0.001).

**Table 5:** Surgical characteristics of lobectomy cases

	Thoracotomy *n* = 8699, 28%	VATS + mini-thoracotomy *n* = 8389, 27%	Complete VATS *n* = 13,970, 45%	*P *value (ANOVA or Kruskal–Wallis test)	*P *value* (Thoracotomy vs VATS + mini-thoracotomy)	*P *value* (Thoracotomy vs complete VATS)
Tumor diameter (cm) mean ± SD	2.02 ± 0.57	1.98 ± 0.57	1.97 ± 0.57	< 0.001	*p* < 0.001	*p* < 0.001
Ope time (min) mean ± SD	214 ± 82	195 ± 71	208 ± 70	< 0.001	*p* < 0.001	*p* < 0.001
Postoperative Hospital stay (day) median, IQR	9 (7–13)	9 (7–12)	8 (6–11)	< 0.001	< 0.001	< 0.001
Blood loss (ml) median, IQR	67 (25–156)	45 (10–100)	50 (10–100)	< 0.001	< 0.004	*p* < 0.001
No. of staplers	5.5 ± 2.2	5.6 ± 2.0	5.9 ± 2.2	< 0.001	0.057	*p* < 0.001
Conversion to thoracotomy	NA	192 (2.3%)	44 (0.31%)	< 0.001**		

*IQR* Interquartile range, *Ope time* operation time

*Post hoc test by multiple comparison with Bonferroni correction

**Fisher’s exact test

Table 6 lists the post-lobectomy complications. The incidence of respiratory failure was lower in the complete VATS group (0.2%) than in the thoracotomy (0.6%) or the VATS + mini-thoracotomy (0.3%) groups. Prolonged air leakage and arrhythmia occurred significantly less in the VATS + mini-thoracotomy group (3.3%, 1.2%) than in the thoracotomy group (4.4%, 2.0%) or the complete VATS group (4.3%, 1.6%). The operative mortality rates in the complete VATS group (0.3%) and the VATS + mini-thoracotomy group (0.4%) were significantly lower than that in the thoracotomy group (0.8%) (*P* < 0.001, *P* < 0.002).

**Table 6:** Major complications of lobectomy

	Thoracotomy	VATS + mini-thoracotomy	Complete VATS	*p* value		
				Thoracotomy vs VATS + mini-thoracotomy	Thoracotomy vs complete VATS	VATS + mini-thoracotomy vs complete VATS
Respiratory failure	51 (0.6%)	29 (0.3%)	28 (0.2%)	0.025	< 0.001	0.039
Pneumonia	214 (2.5%)	101 (1.2%)	155 (1.1%)	< 0.001	< 0.001	0.518
Acute exacerbation of interstitial pneumonia	53 (0.6%)	30 (0.4%)	48 (0.3%)	0.018	0.003	0.863
Prolonged air leakage	379 (4.4%)	277 (3.3%)	597 (4.3%)	< 0.001	0.763	< 0.001
Pyothorax	62 (0.7%)	52 (0.6%)	78 (0.6%)	0.511	0.162	0.588
Broncho pleural fistula	20 (0.2%)	12 (0.1%)	31 (0.2%)	0.218	0.891	0.212
Pulmonary embolus	11 (0.1%)	1 (0.0%)	14 (0.1%)	0.006	0.546	0.014
Cardiac failure	19 (0.2%)	13 (0.2%)	5 (0.0%)	0.379	< 0.001	0.005
Arrhythmia	174 (2.0%)	101 (1.2%)	218 (1.6%)	< 0.001	0.015	0.032
Myocardial infarction	4 (0.0%)	1 (0.0%)	4 (0.0%)	0.375	0.493	0.657
Cerebral infarction, hemorrhage	42 (0.5%)	26 (0.3%)	49 (0.4%)	0.089	0.13	0.632
Chylothorax	65 (0.7%)	69 (0.8%)	121 (0.9%)	0.603	0.364	0.762
Re-operation	14 (0.2%)	16 (0.2%)	22 (0.2%)	0.716	1	0.62
Operative death	68 (0.8%)	34 (0.4%)	48 (0.3%)	0.002	< 0.001	0.495

## Discussion

The number of early lung cancers being diagnosed is increasing with the widespread use of chest computed tomography (CT). Therefore, an analysis of the current status of surgical treatments for stage IA lung cancer based on the NCD data will be highly beneficial. Our analysis of 47,921 clinical stage IA lung cancer patients shows the current status of surgical treatments for early stage lung cancer in Japan. This is because the number of lung cancer operations recorded in the NCD accounts for more than 94% of all cases registered in the Regional Bureau of Health and Welfare in Japan [4, 5]. MIA was the procedure selected for 74.6% of patients, which shows that complete VATS or at least MIA with the use of VATS has become the standard treatment for clinical stage IA lung cancer cases in Japan.

The overall rate of intraoperative injury of the pulmonary artery was 0.85%, occurring in 1.9% of patients who underwent thoracotomy and 0.48% of those who underwent MIA, suggesting the safety of VATS. However, the specific causes of the higher rate of vessel injury in thoracotomy are unclear. We suspect that vessel injuries during MIA and conversion to thoracotomy registered as thoracotomy are underreported.

The definition of “re-operation” in the case report form in the NCD is “redo surgery within 24 h after the initial surgery”. This may account for the smaller number of recorded cases of re-operation than the actual number of cases. An inherent limitation to the current NCD is the lack of a slot for the actual number of cases. Thus, the actual number of redo-operations could not be analyzed.

Although the reasons for MIA conversions to thoracotomy were not stated in the NCD registry, vessel injury, severe adhesion, and incomplete interlobular fissure were suspected. The rates of conversion from VATS + mini-thoracotomy and complete VATS were 2.1% and 0.3%, respectively. There may be a slight difference in the policy of selecting the surgical approach depending on the institution and the surgeon; however, the reasons for the difference in the conversion rates could not be clarified in the current study. In the future, we would like to compare the changes in surgical outcomes over time according to regions, to identify areas for improvement.

The mean operation time was shorter and the mean blood loss was less in the MIA group than in the thoracotomy group. Scott et al. published similar results indicating that the median operation time was significantly shorter in a VATS group than in a thoracotomy group (117.5 min vs. 171.5 min, *P* < 0.001) in their propensity-matched analysis [10]. A systematic review revealed two types of reports, one showing that VATS lobectomy prolonged the operation time and another showing that the operating time did not significantly differ between the approaches [11]. The mean number of staples used was five for both thoracotomy and MIA. As the NCD does not have entry slots for a rapid pathological diagnostic procedure, the number of staples used for wedge resections for this purpose cannot be identified separately.

The postoperative mortality and morbidity rates were lower in the MIA group than in the thoracotomy group, in accordance with the previous reports [12, 13]; however, the underlying reason remains to be clarified. Gopaldas et al. reported similar postoperative morbidity rates after thoracotomy (*n* = 12,860) and VATS lobectomy (*n* = 759) [14]. On the other hand, Scott et al. reported significantly fewer patients with complication in the VATS group than in the thoracotomy group (27.3% vs 47.8%, *P* < 0.005) [10]. Propensity-matched analysis should be performed to clarify the lower morbidity after VATS more accurately [15].

Recently, Japanese surgeons have been able to perform curative operations for early lung cancer using MIA, especially with the development of advanced instruments and imaging techniques. However, some conservative surgeons still consider thoracotomy better for avoiding vessel injury and thus, have not embraced VATS. Pathological stage II and IIIA disease (up-staging) was observed more frequently in the thoracotomy group than in the MIA group (11.8% vs 7.3%). It is possible that lymph node dissection was more systemic in the thoracotomy group, or that very early lesions tended to be treated by MIA. However, these were only speculations.

Our study was limited by the fact that it focused only on obtaining a representative picture of the surgical treatments for early stage lung cancer in Japan rather than on analyzing comparative effectiveness. However, we acknowledge that the choice of surgical approach is also subject to potential selection bias or operator/center experience. Further analysis of the differences in the frequency of thoracotomy and VATS, as well as in the frequency of lung cancer surgery using MIA among institutions would be important components of our subsequent studies. In the future, we intend to compare changes in surgical outcomes over time and explore the management of a more heterogeneous group, such as patients with stage IIIa disease, that would benefit from a large number of patients in the NCD. We will also consider clarifying primary endpoints and their multivariable analyses. At this stage, the NCD does not include prognostic data; therefore, the relationship between MIA and prognosis cannot yet be analyzed.

In conclusion, our analysis of the surgical treatments for 47,921 clinical stage IA lung cancer patients in Japan indicated that MIA using the VATS technique has become the new standard treatment for early lung cancer. As this study analyzed objective “real world” data from a nationwide database, it presents valuable information for further clinical research on minimally invasive surgery.

## Electronic supplementary material

Below is the link to the electronic supplementary material.

Supplementary file1 (DOCX 17 kb)
