# Supplementary material for: Current status of surgery for clinical stage IA lung cancer in Japan: analysis of the national clinical database
Source: Surg Today. 2020 Jul 5;50(12):1644–51. doi: 10.1007/s00595-020-02063-x (PMC7677152; doi:10.1007/s00595-020-02063-x)
Supplement: Supplementary file 1 — Supplementary file1 (DOCX 17 kb) [file 595_2020_2063_MOESM1_ESM.docx]

**Supplemental table 1. Data items for lung cancer surgery**

**Preoperative information**

Height, weight, BMI

Performance status

Smoking history

Pulmonary function test

Anticancer treatment (if the disease is a thoracic malignancy)

Comorbidity

　 Coronary artery disease

Anticancer surgery within the past 5 years

Disease of the central nervous system (required treatment)

Diabetes mellitus (required treatment)

Hemodialysis

Liver dysfunction (Child-Turcotte classification B or C)

Anemia (Hb ≤ 8.0 g/dl)

Interstitial pneumonia

Autoimmune disease (required treatment)

Clinical stage (TNM)

Maximum tumor diameter

**Operative information**

Operative time

Blood loss

Approach (Thoracotomy, VATS, Robot)

Number of staples

Energy device

Fibrin glue

Extracorporeal circulation

Intraoperative transfusion

Intraoperative injury

Procedure (main procedure, nodal dissection combined resection, curability)

Detailed VATS approach

Number of access ports

Maximum length of skin incision

Conversion to thoracotomy

**Postoperative information**

Pathological stage (TNM)

Histology

Maximum tumor diameter

Postoperative complication

Prolonged air leakage (> 6 days)

Atelectasis (required bronchoscopy)

Pneumonia

Acute exacerbation of interstitial pneumonia

Respiratory failure (Ventilation support > 48 hours)

ARDS

Bronchopleural fistulae

Pulmonary emboli

Arrhythmia (required treatment)

Myocardial infarction

Congestive heart failure (required treatment)

Cerebral hemorrhage

Cerebral infarction

Hoarseness

Delirium (required treatment)

Empyema (required drainage)

Mediastinitis (required drainage)

Wound infection (required drainage)

Redo-surgery

Bleeding (required blood transfusion)

Chylothorax

Renal failure (required hemodialysis, creatinine > 4 mg/dl)

Liver failure (required treatment)

VATS, video-assisted thoracic surgery
